# An assessment of enhanced biosecurity interventions and their impact on porcine reproductive and respiratory syndrome virus outbreaks within a managed group of farrow-to-wean farms, 2020–2021

**DOI:** 10.3389/fvets.2022.952383

**Published:** 2023-01-12

**Authors:** Karyn A. Havas, Lisa Brands, Roger Cochrane, Gordon D. Spronk, Joel Nerem, Scott A. Dee

**Affiliations:** ^1^Pipestone Research, Pipestone Holdings, Pipestone, MN, United States; ^2^Pipestone Nutrition, Pipestone Holdings, Pipestone, MN, United States; ^3^Pipestone Veterinary Services, Pipestone Holdings, Pipestone, MN, United States

**Keywords:** porcine reproductive and respiratory syndrome virus (PRRSV), biosecurity, feed mitigation, air filtration, swine

## Abstract

**Introduction:**

Porcine reproductive and respiratory syndrome virus (PRRSV) has been a challenge for the U.S. swine industry for over 30 years, costing producers more than $600 million annually through reproductive disease in sows and respiratory disease in growing pigs. In this study, the impact of enhanced biosecurity practices of site location, air filtration, and feed mitigation was assessed on farrow-to-wean sites managed by a large swine production management company in the Midwest United States. Those three factors varied in the system that otherwise had implemented a stringent biosecurity protocol on farrow-to-wean sites. The routine biosecurity followed commonplace activities for farrow-to-wean sites that included but were not limited to visitor registration, transport disinfection, shower-in/shower-out procedures, and decontamination and disinfection of delivered items and were audited.

**Methods:**

Logistic regression was used to evaluate PRRSV infection by site based on the state where the site is located and air filtration use while controlling for other variables such as vaccine status, herd size, and pen vs. stall. A descriptive analysis was used to evaluate the impact of feed mitigation stratified by air filtration use.

**Results:**

Sites that used feed mitigates as additives in the diets, air filtration of barns, and that were in less swine-dense areas appeared to experience fewer outbreaks associated with PRRSV infection. Specifically, 23.1% of farms that utilized a feed mitigation program experienced PRRSV outbreaks, in contrast to 100% of those that did not. Sites that did not use air filtration had 20 times greater odds of having a PRRSV outbreak. The strongest protective effect was found when both air filtration and feed mitigation were used. Locations outside of Minnesota and Iowa had 98.5–99% lesser odds of infection as well.

**Discussion:**

Enhanced biosecurity practices may yield significant protective effects and should be considered for producers in swine-dense areas or when the site contains valuable genetics or many pigs.

## 1. Introduction

Porcine reproductive and respiratory syndrome virus (PRRSV) is a positive-sense RNA virus in the *Arteriviridae* family and is classified into two genotypes *Betaartirivirus suid 1* and *2*, the historically European and North American PRRSV variants, respectively (1). Since its emergence in the late 1980s and isolation in the 1990s (2, 3), the economic impact of PRRSV on the swine industry was substantial and has been estimated to cost $560 million in 2005 and $664 million in 2013 in breeding and growing pig herds in the United States (4, 5). The clinical presentation has included reproductive failure in gilts and sows, as well as respiratory disease and mortality in growing pigs (6, 7). Between 2009 and 2013, 33–38% of farms representing 21% of the industry's production were infected with PRRSV (8), speaking to the high burden of disease in the industry. This disease burden has persisted with a cumulative incidence of >20% of farms in the United States experiencing outbreaks annually (9). This incidence rate coupled with the significant genetic diversity and rates of mutation (10, 11) has made control difficult, through traditional strategies such as vaccination (12–14).

Despite the severity of the clinical disease, it is known that biosecurity practices are effective at limiting infection with PRRSV (15, 16). PRRSV is transmitted between sites in a variety of ways, including the movement of infected pigs onto the site, use of infected semen in sows, use of contaminated trucks, ingestion of contaminated feed, and aerosol transmission between farms (17). Clearly, routine biosecurity practices remain critical to disease control. Yet, new scientific discoveries uncover new pathways of transmission that require biosecurity interventions. Two interventions, air filtration and feed mitigation, have been included in the concept of “Next Generation Biosecurity” (18) and are meant to tackle more recent biosecurity challenges.

It has only been since the outbreak of the porcine epidemic diarrhea virus in the United States in 2013 that feed as a carrier vehicle for viral pathogens has been established (19, 20). Yet, since then there has been overwhelming evidence that pathogens, including PRRSV, survive in feed and pigs can be infected following consumption of contaminated feed (21, 22). In an effort to mitigate this threat, a variety of feed additives, such as acids or formaldehyde solutions, have been experimentally evaluated and found effective at reducing PRRSV transmission to pigs in feed (23) after multiple experimental studies showed their effectiveness in reducing the risk of porcine epidemic diarrhea virus transmission, as well as several other viruses (24–27). These additives will be classified as feed mitigants throughout this study.

Furthermore, it has also been thoroughly documented that PRRSV has documented aerosol spread (28, 29) of ranges up to 9.1 km (23). A series of experiments showed the effectiveness of air filtration in preventing transmission of PRRSV between chambers (30) and between barns housing PRRSV-infected pig populations (31, 32). Air filtration was confirmed to be effective on operational farms through a retrospective case–control study conducted in a swine-dense region (33) when using mechanical air filtration.

Studies evaluating swine density have shown that increased density leads to an increased risk of PRRSV-associated outbreaks (34–36). In addition, swine-dense regions also show an increased risk of PRRSV outbreaks in the summer (36). There are also numerous other factors that can contribute, such as terrain, vegetation, and altitude (34, 37); therefore, the location of a site can play a role in disease transmission.

The objective of this study was to evaluate the efficacy of enhanced biosecurity tools of location for future production sites, feed mitigants, and air filtration on the risk of PRRSV infection in a large swine production system in the Midwest United States. This study will provide further guidance to swine production stakeholders as to what biosecurity protocols provide enhanced protection to their businesses.

## 2. Methods

### 2.1. Data description and sources

Pipestone Management and Pipestone Veterinary Services (Pipestone) (https://www.pipestone.com/services/swine/) provides swine production and veterinary medical care oversight to farrow-to-wean sites across eight states in the mid-West and upper mid-West of the United States. All farrow-to-wean sites managed from January 2020 to January 2022 by Pipestone that had either a wild-type variant outbreak or no outbreak were included in this study. Any site that had a vaccine variant-associated break was removed. Wild-type variants associated with outbreaks were defined as outbreaks whose viral variant sequence and restriction fragment length polymorphism (RFLP) pattern was not homologous with vaccine strains, commonly the 2-5-2 RFLP pattern, as it is known to be associated with vaccine strains (38). Diagnostic samples analyzed by regional veterinary diagnostic laboratories in the United States at the time of the outbreak provided PRRSV real-time PCR results (positive/negative), open reading frame 5 (ORF5) sequences, and RFLP patterns. If a site experienced multiple outbreaks, additional outbreaks were only included if they were caused by a different variant based on RFLP patterns and ORF5 sequencing and had at least one calendar year between outbreaks; two sites had multiple outbreaks evaluated based on these criteria. This was implemented since sites could modify and enhance their biosecurity over time and PRRSV outbreaks impacted biosecurity and processes at these sites as well. A similar method had been used previously (39). In total, 69 observations from 67 sites were evaluated.

Management and farm characteristics data were available from the farrow-to-wean farm sites enrolled in Pipestone Management. Data used were collected from routine management and veterinary procedures of these sites and included information collected from Pipestone Management records, production records, and veterinary records. County-level hog inventory data were obtained through Quick Stats using the 2017 USDA Agricultural Census data (40) and are included in Table 1.

**Table 1:** Summary of PRRSV-infected farrow-to-wean farm characteristics and associated univariable analysis in the Pipestone Management System from January 2019 to January 2022.

	**Overall**	**PRRSV outbreaks** **(*n =* 30)**	**No PRRSV outbreaks** **(*n =* 39)**	**Univariable** ***p*-value**
	**Median (range)**	**Median (range)**	**Median (range)**	
**Herd size**	3,609 (1,362, 9,712)	3,184 (1,362, 7,139)	5,135 (1,397, 9,712)	0.014
County swine density^*^	96,226 (>0^†^, 1,072,839)	274,059 (179, 1,072,839)	67,238 (0^†^, 367,983)	Not assessed
**Primary air filter age (mos)**	33.5 (3.9, 73)	24.4 (8.8, 73)	34.2 (3.9, 61)	0.686
**Secondary air filter age (mos)**	12.2 (0.2, 24)	11.2 (0.2, 24)	12.2 (3.9, 13.6)	0.33
	# (%)	# (%)	# (%)	# (%)
**Air filter use**	42 (60.9%)	13 (43.3%)	29 (74.4%)	0.01
**Stall-housing (vs. Pen)**	51 (73.9%)	25 (83.3%)	26 (66.7%)	0.124
PRRSV health status (prior to outbreak for sites that had an outbreak)^**^				0.0182
Negative	1 (1.5%)	0 (0%)	1 (1.5%)	
Stable herd infection without vaccine	31 (44.9%)	8 (26.7%)	23 (59.0%)	
Stable herd infection with vaccine	33 (47.8%)	18 (60.0%)	15 (38.5%)	
Unstable herd infection	0 (0%)	0 (0%)	0 (0%)	
Outbreak	4 (5.8%)	4 (13.3%)	0 (0%)	
**PRRS vaccination use**	37 (53.6%)	22 (73.3%)	15 (38.5%)	0.005
**State of site location**				<0.001
Iowa	15 (21.7%)	13 (43.3%)	2 (5.1%)	*Reference*
Minnesota	15 (21.7%)	9 (30%)	6 (15.4%)	0.112
Other	15 (21.7%)	3 (10%)	12 (30.8%)	0.001
South Dakota	24 (34.8%)	5 (16.7%)	19 (48.7%)	0
**PRRS RFLP patterns**	30			
1-2-4	2 (6.7%)			
1-3-2	3 (10%)			
1-3-4	3 (10%)	Grouped into 2 different sequence homologies		
1-7-4	6 (20%)	Grouped into 3 different sequence homologies		
1-8-4	5 (16.7%)	Grouped into 3 different sequence homologies		
1-4-4^***^	10 (33.3%)	Grouped into 3 different sequence homologies		
1-4-2	1 (3.3%)	Sequence homology aligned with 1-4-4 lineage 1C		

* Not included in the multivariable analysis due to small sample size and wide range of values.

** Variable categories were collinear, and the overall variable was excluded from the regression model.

*** Six isolates were 1-4-4 lineage 1C with similar sequence homologies.

† County density data are from the 2017 USDA Agricultural Census, which measured the county density at zero. The county now has a Pipestone-managed swine farm.

Farm characteristics included health status level based on PRRSV status and vaccine use (41), herd size, air filter usage, air filter age, pen vs. stall usage, and feed mitigant usage. Feed mitigants used in the system included Guardian^TM^ (Alltech, Nicholasville, Kentucky, USA), Sal CURB® (Kemin Industries, Des Moines, Iowa, United States), and Activate^TM^ (Novus International, Saint Charles, Missouri, United States). Sal CURB® was used if the feed mill had the correct set-up and equipment to use it, Guardian^TM^ was used if Sal CURB® was not able to be used, and Activate^TM^ was used sporadically when Guardian^TM^ was not available. Sal CURB® was a liquid, formalin-based feed mitigant, and Guardian ^TM^ and Activate ^TM^ were organic acid-based. Air filtration used MERV14 filters as a primary filter followed by MERV8 pre-filters for secondary filtration when used. Filtration styles varied with some being attic-based and others being side and that information was not captured. All facilities employed a negative pressure ventilation design that was inspected daily by a filtration compliance technician.

### 2.2. Summary of farm characteristics in the system

Table 1 summarizes the site-level observations from January 2020 to January 2022, except for feed mitigant data which were evaluated between November 2020 and January 2022. The system originally had 70 observations from 67 farrow-to-wean sites. One site broke three times in this period with different wild-type variants, first with a 1-4-4 variant in May 2020, a second time with a 1-7-4 variant in June 2021, and a third time with a 1-8-4 variant in September 2021; the variants had less than a 90% sequence homology of the ORF5 region. The September 2021 sequence was excluded from further analyses since it occurred within 1 year of the other outbreaks, leaving 69 total observations for further analysis. Another farm broke twice, once with a 1-3-4 variant in November 2020 and again with a 1-7-4 variant in January 2022. Both outbreaks were kept since the sequence homology was 87%. This left 69 observations at 67 sites from January 2020 to January 2022. There were 30 with PRRSV outbreaks from 28 (41.2%) sites, and these were compared to 39 sites that did not have wild-type PRRSV outbreaks. Overall, there were 42 (60.9%) sites that installed mechanical air filtration with a median primary- and secondary-air filter age of 33.5 and 12.2 months, respectively. These sites had air filters for the duration of the study period. Three sites that used primary filters did not use secondary filters. Most sites used to stall or crate housing (51, 73.9%). The overall median farm size was 3,609 sows. Most sites had stable PRRSV infections with or without vaccination (64, 92.7%), and 37 sites (53.6%) conducted PRRSV vaccination. All 2-5-2 RFLP patterns had common sequence homologies, were related to vaccines, and were not included in this analysis. Only wild-type variant-associated outbreaks were assessed. There were 15 sites (22.4%) each in Iowa and Minnesota, and the remaining 37 were in other states (55.2%; Indiana, Illinois, Wisconsin, Missouri, and North Dakota), with most sites being found in South Dakota (24, 35.8%). Infected sites were found in Iowa (13/15), Minnesota (9/15), South Dakota (5/24), Missouri (1/4), and Illinois (2/7). Indiana (1 site), North Dakota (1 site), and Wisconsin (2 sites) did not have any infected sites (see Table 1).

An ethical review of the study was not needed as data were used retrospectively after outbreaks had naturally occurred on sites raising pigs for the purpose of sale for pork production. Pipestone has permission to use site data for research and publication as part of the management contracts.

### 2.3. Study design and statistical analyses

A case-control study compared enhanced biosecurity measures while controlling for and evaluating farm characteristics between sites that experienced a PRRSV outbreak and sites without a PRRSV outbreak between January 2020 and January 2022 in the Pipestone Management System. Data were available at the time of each outbreak and for the infected sites, and they were compared to the most current data for the sites that did not experience an outbreak. Cases were outbreaks by wild-type variants at the farrow-to-wean sites. Controls were any site in the system that did not experience a PRRSV outbreak during this period.

Farm characteristics evaluated as explanatory variables for PRRSV outbreaks were summarized using descriptive statistics. The logistic regression model was built and evaluated following procedures described by Dohoo et al. (42) using the following protocol. Univariable logistic regression of each of the categorical explanatory variables against the site PRRSV status was done for initial variable selection with a level of significance (α) set at 0.2. It was found the PRRSV health status categories were collinear, and this was exacerbated by the presence of PRRSV vaccine use. Since PRRSV vaccine use is related to the PRRSV health level and since it had a smaller *p-*value in the univariable analysis, it was kept in the model and the PRRSV health levels were dropped. Variables for the final model were chosen using backward variable selection and a level of significance of 0.05. The final model was evaluated for overall significance at a level of 0.05 and for the goodness of fit using the Hosmer–Lemeshow and Pearson chi-squared tests with a level of significance of 0.05. Due to small sample sizes, confounding was not further assessed to prevent introducing further bias into the model (43). Analysis was performed using STATA 16.1 IC (Stata Corp, College Station, Texas), and data management was done using Microsoft Excel version 16.63.1 (Microsoft Corporation, Redmond, Washington).

Feed mitigation use varied throughout the year, unlike the other variables considered, and not all sites had adequate data. Therefore, feed mitigation was not included in the regression model as it would have further reduced the number of observations and could not be accurately represented. Instead, a descriptive summary was completed. The incidence risks of PRRSV outbreaks per 1,000 site-days on-feed mitigants (exposed) and off-feed mitigants (unexposed) for each site and the system overall were calculated from 1 November 2020 to 8 January 2022. The system incidence risk compiled the number of PRRSV breaks across all days that sites in the system were on-feed mitigants and off-feed mitigants and developed one summary measure, while site incidence risks were compiled for each site and were summarized by median and range. In this way, sites were compared to themselves. Site and system incidence risks were also stratified across site air filtration use status. Four sites did not order feed through Pipestone, thus, data were not available on the timing of their feed mitigant use (*n* = 63). Additive and multiplicative negative (protective) interactions between system incidence risk stratified by air filtration and feed mitigation use were evaluated and assessed using equations described by VanderWeele (44). Since a small number of infections occurred (19 overall), site-level incidence comparison was not performed.

## 3. Results

### 3.1. Retrospective case-control study on sow sites

This analysis compared outbreaks between PRRSV-infected and uninfected sites. There were 69 observations from 67 sites included in this analysis. The univariable analysis resulted in the inclusion of herd size, air filtration, housing type, PRRSV vaccination use, and location (US state) being included in the maximum multivariable model (Table 1). The final logistic regression model included air filtration use and the state where the site was located (see Table 2). Sites that did not use filtration had 20 times greater odds (*p*-value = 0.006) of being infected compared to those that did not when the state was controlled. The use of air filtration reduced the predicted probability of being infected by 24.9% in Iowa, 41.6% in Minnesota, 40.9% in South Dakota, and 36.2% in all other states. Sites in Iowa had 100 times the odds of being infected than sites in South Dakota as well as sites in Illinois, Indiana, Missouri, North Dakota, and Wisconsin (other categories). The baseline odds of being infected (an unfiltered site in Iowa) were 56.2 greater.

**Table 2:** Logistic regression coefficients and odds ratio in comparing PRRSV-infected sites to uninfected sites in the Pipestone Management System from January 2020 to January 2022.

	**Final model**			
**Predictors**	**Coefficient**	**Odds ratio**	**OR 95% confidence interval**	***P*** **-value**
**Filter use**				0.006
No	*Reference*			
Yes	−3.01	0.05	(0.006, 0.42)	
**State**				<0.0001
IA	*Reference*			
MN	−0.84	0.43	(0.06, 2.99)	0.396
Other	−4.46	0.01	(0.001, 0.175)	0.001
SD	−4.24	0.01	(0.001, 0.178)	0.001
**Baseline**	4.03	56.2	(4.51, 699.88)	0.002
**Model significance**				<0.0001
**State/air filtration**	**Predicted probability**		**95% confidence interval**	
IA/unfiltered	98.3%		(93.9%, 100%)	
IA/filtered	73.4%		(42.0%, 100%)	
MN/unfiltered	96.1%		(87.1%, 100%)	
MN/filtered	54.5%		(27.7%, 81.2%)	
SD/unfiltered	44.7%		(15.2%, 74.1%)	
SD/filtered	3.8%		(0%, 11.6%)	
Other/unfiltered	39.3%		(4.9%, 73.8%)	
Other/filtered	3.1%		(0%, 10.1%)	

The Hosmer–Lemeshow *p*-value was 0.6056, and the Pearson chi-squared *p*-value was 0.3056.

### 3.2. Descriptive summary of feed mitigants

This analysis was from data obtained between November 2020 and January 2022 and included information from 63 sites with complete feed mitigation use data. Table 3 describes this assessment. Only two sites did not use feed mitigants at any point during this period, and one of them was in Iowa and had a wild-type PRRSV break. Not all sites that used mitigants used them continuously during the analysis period. Only one site used Sal CURB (1.6%) (formaldehyde-based mitigant) and was in Minnesota, filtered, and never experienced an outbreak. They used it continuously from before the study period began. Nine (9.5%) other sites began using organic acid mitigants (Guardian and Activate) continuously starting in the winter of 2020 to 2021 after the analysis began. Of these nine, six (66.7%) were filtered and were in Minnesota (4), South Dakota (1), and the other (1) states. Three outbreaks occurred on these sites after starting mitigants, one in Iowa and two in Minnesota. Another outbreak occurred on the same Iowa site in November 2020, before it started using mitigants. There were 19 PRRSV outbreaks, eight on sites that were using feed mitigants at the time of the break, and 11 when feed mitigants were not being used (see Table 3).

**Table 3:** Descriptive summary of PRRSV outbreaks, as well as feed mitigant and filter used by farrow-to-wean farms in the Pipestone Management System from November 2020 to January 2022.

	**#**	**Filtered**	**Filtered sites by state**
**Total sites included**	63	41 (65.1%)	IA (6), MN (13), SD (14), Other (8)
Sites that used mitigants at some point	61	41 (67.2%)	IA (6), MN (13), SD (14), Other (8)
Sites that never used mitigants	2	0 (6%)	IA and Other
Sites that continuously used mitigants since before November 2020	1	1 (100%)	MN
Sites that continuously used mitigant after starting in fall/winter 2020–2021, after November 2020	9	6 (66.7%)	MN (4), Other (1), SD (1)
**Feed mitigant**	**Overall**	PRRSV outbreaks^*^	**No PRRSV outbreaks**
No mitigant used	2 (3.2%)	11 (57.9%)	6 (13.3%)
SalCurb	1 (1.6%)	0 (0%)	1 (2.2%)
Guardian/activate	60 (95.2%)	8 (42.1%)	38 (84.4%)
	Median days used (IQR^**^)		**Outbreaks**
Mitigant used	276 (119, 386)		8
Mitigant not used	162 (57, 310)		11
Total	63	443 (437, 443)	

* One site had a PRRSV infection when using feed mitigants and when not.

** Interquartile range provides the value at the 25th percentile and 75th percentile.

When calculating system and site-level incidence risk for PRRSV outbreaks only farms that varied their feed mitigant use were included to allow for accurate comparisons, this reduced the sites included to 60 as one farm used a mitigant continuously and two never used a mitigant at all (Table 4). The overall system-level incidence risk of PRRSV outbreaks per 1,000 days when using mitigants was 0.5 compared to 1.1 per 1,000 site-days when not [incidence risk ratio (IRR) = 0.45]. When air filters were not used, the system-level incidence risk of PRRSV outbreaks when not using a mitigant rose to 1.8 per 1,000 site-days and the IRR for mitigant vs. non-mitigant use was 0.22. When using air filtration, the system-level incidence risk of PRRSV outbreaks when using feed mitigant use was 0.5 per 1,000 site-days and was 0.7 per 1,000 site-days (IRR 0.71) when not using a feed mitigant. Without mitigants, when using an air filter, the system-level incidence risk of PRRSV outbreaks was 0.7 per 1,000 site-days. Filtration and feed mitigation are purely additive, and there is no statistical interaction between the interventions (44). As for site-level incidence risk per 1,000 site-days, the median risk across all sites was zero breaks, and the differences between sites were seen in the maximum of the ranges. Sites that used feed mitigants had a lower maximum number of breaks per site in 1,000 site-days compared to sites that did not use feed mitigants: 5.7 and 12.5 overall, 3.4 and 12.5 when air filters were not used, and 5.7 and 8.1 when filters were used, respectively. The sites that did not use feed mitigants but used air filters had a range maximum of 8.1 compared to sites that did not use air filters or mitigants, which had a range maximum of 12.5 per 1,000 site-days.

**Table 4:** Incidence of PRRSV infection by 1,000 site-days stratified by feed mitigant and filter use among sites that varied their feed mitigant use in the Pipestone management system from November 2020 to January 2022.

**Sites included in the risk calculation**, ***n*** = **60**	**Summary of incidence risk per 1,000-site days**		
	**System incidence risk**	**Median incidence risk for all sites (range)**	**System incident risk ratio**
Incidence risk with feed mitigants	0.5	0 (0, 5.7)	0.45
Incidence risk without feed mitigants	1.1	0 (0, 12.5)	
**Did not use air filters**			
**Sites included in the risk calculation**,	**System incidence risk**	**Median incidence risk for**	**System incident risk ratio**
***n*** = **20**		**all sites (range)**	
Incidence risk with feed mitigants	0.4	0 (0, 3.4)	0.22
Incidence risk without feed mitigants	1.8	0 (0, 12.5)	
**Used air filters**			
**Sites included in the risk calculation**,	**System incidence risk**	**Median incidence risk for)**	**System incident risk ratio**
***n*** = **40**		**all sites (range**	
Incidence risk with feed mitigants	0.5	0 (0, 5.7)	0.71
Incidence risk without feed mitigants	0.7	0 (0, 8.1)	
**Did not feed mitigants**			
	**System incidence risk**	**Median incidence risk for**	**System incident risk ratio**
		**all sites (range)**	
Incidence risk with air filters, *n =* 40	0.7	0 (0, 8.1)	0.39
Incidence risk without air filters, *n =* 20	1.8	0 (0, 12.5)	
**Protective additive interaction**	R_mf_ < R_m0_ + R_0f−_ R_00_^*^	0.5 < (0.4 + 0.7–1.8) < −0.7	False
**Protective multiplicative interaction**	R_mf_ < (R_m0_ * R_0f_)/R_00_^*^	0.5 < (0.4*0.7)/1.8 < 0.15	False

* Equations from VanderWeele (44) were used to determine whether there were negative or protective interactions. R_mf_ is the system incidence risk when both feed mitigation and air filtration were used. R_m0_ is the system incidence risk when feed mitigation was used but air filtration was not. R_0f_ is the system incidence risk when feed mitigation was not used but air filtration was used. R_00_ is the system incidence risk when feed mitigation and air filtration were not used.

## 4. Discussion

Porcine reproductive and respiratory syndrome virus is an economically damaging disease to swine producers. As a positive-sense RNA virus, it undergoes regular mutation (10, 11) and impacts US swine sites every year (8). The intent of this study was to assess the enhanced biosecurity protocols that use air filtration, feed mitigation, and have sites in different states and their impact on PRRSV infections in a large U.S. swine system from 2020 to 2021. The results demonstrated the protective nature of location by state and air filtration. It also exhibited that there may be a protective effect when using feed mitigation if these enhancements are layered on a well-established biosecurity program.

This study demonstrated the association between the use of air filters and the reduction in PRRS outbreaks. In this study, the only biosecurity and/or risk components in the system that varied by site were location by state, use of air filtration, and feed mitigation. All other routine biosecurity measures were implemented through standard operating procedures and ongoing training. They were evaluated monthly through a standardized audit program. These procedures included shower-in/shower-out, visitor control, disinfection and decontamination of incoming supplies, downtime for veterinarians before going to farrow-to-wean farms, truck wash requirements before coming to the farm, feed shipment schedules to reduce contamination, management and composting of mortality, and others. Such a program would likely be a critical component to have in place to achieve similar results as was seen in this study with air filtration and feed mitigation. Furthermore, the movement of weaned piglets off healthy farrow-to-wean farms sites occurs routinely on a bi-weekly basis for all farms using Pipestone-managed transportation which employs mandatory downtime and truck washes between sites. Receipt of replacement gilts is done using appropriate quarantine and testing procedures, and semen is tested before delivery each week.

Air filtration played a significant role in biosecurity as farms that did not use air filtration had 20 times greater odds of infection when the location was controlled for in the analysis. These findings supported other studies that have shown that mechanical air filtration was effective at reducing PRRSV transmission (30, 31, 33), but it cannot reduce risk to zero. The air filtration program implemented in this study was standardized across all participating farms, specifically using MERV14 fiberglass mechanical filters in conjunction with negative pressure ventilation. On every participating farm, a designated individual, the filtration compliance technician, oversees the integrity of the air filtration program daily throughout the study. The location also played a significant role. When air filtration was present at a site in Iowa, the estimated probability of infection went from 98.2 to 73.7%. Compare this to the estimated probability of an outbreak without air filtration in South Dakota (44.5%) to the probability of an outbreak with air filtration (3.9%). In both cases, air filtration substantially reduced the risk of an outbreak, but it was also clear that the starting probability of an outbreak without air filtration differed significantly, with sites in South Dakota having less than half the risk of sites in Iowa. The combination of air filtration and location was highly protective.

Feed mitigation also may help prevent PRRSV-associated outbreaks in this system. The use of a feed mitigant decreased the incidence risk by 75% when there was no air filtration and 29% when there was air filtration. Numerous experimental studies have shown that feed can be contaminated and transmit the PRRSV virus (15–23), and another experimental study has shown that feed mitigants can ameliorate that risk to some extent (23). In this study, we found that sites that did not use a mitigant had a higher incidence risk, and year-round mitigant use may reduce risk, although further study is needed. Anecdotally, Pipestone implemented year-round mitigant use for sites in the system that use Pipestone Nutrition starting in the summer of 2021. In the year since, the system has had its lowest PRRSV outbreak cumulative incidence rate to date (7.7%) (unpublished data, Pipestone Research). For comparison, the previous 3 years had rates of 15.1, 18.6, and 30.9% (unpublished data, Pipestone Research). It is substantially lower than the cumulative incidence from the 36 systems that contribute to the Morrison Swine Health Monitoring Program, which was 24.2% for 2021–2022 (45). There are no studies to date on the frequency that PRRSV is found in feed, and such work is needed. There are documented instances when PEDV was found in feed after introduction to sites that received pigs from other negative sites in Mexico (46), in previously negative farrow-to-wean sites in China (47), as well as associated with the first outbreaks in Canada (20). Using feed mitigants may reduce exposure *via* feed, and if respiratory exposure was also limited through air filtration, then multiple sources of infection would be limited by working on two separate pathways of transmission.

It should also be considered what variables were insignificant. Farm size was not significant in this model, yet the location was. This is in contrast with multiple recent publications that have both herd size and swine density as risk factors (34–36). It was reported that herd sizes of greater than 2,500 pigs had an incidence risk ratio of 1.31 (34), while our median farm size overall was 3,609 pigs, for sites with PRRSV outbreaks was 3,184, and for sites without PRRSV outbreaks was 5,135. This study is either constrained by sample size in detecting herd size impacts or the management methods in place in the Pipestone System mitigated the increased risk associated with herd size. Sites that used pen housing for stall housing showed no difference in infection either, although movement spread in the barn was not assessed. Vaccination status was also not significant. This confirmed a previous study that PRRSV vaccination does not prevent infection (48, 49), but other studies have reported that vaccination reduces clinical signs once infected (50–52).

Data used were from a well-established swine production system with a standardized and audited biosecurity program that currently manages approximately 70 farrow-to-wean sites in the United States across eight states that accounts for approximately 300,000 sows. The strength of this study was the use of real-world farm sites and natural infections through which one could retrospectively assess enhanced biosecurity measures that were implemented after a long history of experimental studies. It is the first study of its kind to evaluate the field application of feed mitigation and air filtration in a large commercial production system with a standardized biosecurity program that spans eight states. Furthermore, it included almost all sites in the system, so it was representative of the system's experience. The limitations of the study were the sample size and the evaluation of one management system. Sample size limited our ability to assess areas of granularity below the location level of the state, such as county density. This study was not able to assess site proximity to roads, feed mills, packing plants, fairs, or other high-risk activities in the vicinity. Furthermore, information about neighboring farms is not measured including their biosecurity practices, disease burdens, types of pigs raised, trucking and transportation practices, or other significant considerations that could also explain PRRSV outbreak risks in an area. Such detailed information was not available but could provide a further understanding of how site location is predictive. This analysis revealed that location was an important factor, but no details on why it was an important factor. There are numerous studies highlighting spatial variables that impact PRRSV risks such as density, vegetation, terrain, and altitude (34, 35, 37). Further study on location-level information should be done considering how these variables differ state by state or county by county in swine-dense areas. In addition, a further study that compiles data across production systems should be analyzed to determine whether enhanced biosecurity measures improve outcomes consistently and whether feed mitigant impacts have statistical significance.

Viruses will continue to negatively impact health outcomes and will be costly. In response, biosecurity measures need to be targeted to known transmission pathways to limit their effect on populations of animals. Feed mitigation and mechanical air filtration represent the most recent advances in commercial swine production biosecurity, and the combination of these approaches has been called “Next Generation Biosecurity” (18). Next Generation Biosecurity (NGB) is built upon a strong foundation of routine biosecurity, which must be uniformly implemented and audited to ensure its greatest protective effect. In addition to good on-site biosecurity practices, there are three components that can be included in applying NGB: location, feed mitigation, and mechanical air filtration. The location of a site had the greatest odds of being diseased in the Pipestone System. Building sites or planning new sites in appropriate locations will be a critical component of biosecurity practices. The use of mechanical air filtration was strongly protective and greatly enhanced site-level biosecurity. Successful implementation requires higher-quality equipment and intensive management of the filtration system. Feed mitigation, albeit relatively new, may provide a needed solution to the recently identified risk factor of feed contamination, but further study is needed (48). This study supports findings from the experimental studies on the efficacy of air filtrations and provides initial support to experimental findings regarding feed mitigation efficacy in controlling PRRSV transmission, although more work is needed for the latter intervention. Given the experimental studies and the outcomes of this observational study, we believe that air filtration and feed mitigation should be the next steps in swine industry biosecurity.

## Data availability statement

The data analyzed in this study is subject to the following licenses/restrictions: The data is not publicly available and contains proprietary information. Requests to access these datasets should be directed to karyn.havas@pipestone.com.

## Ethics statement

Ethical review and approval was not required for the animal study because this study used production and medical data generated as part of normal farm operations and not originally meant for research purposes. This study conducted a retrospective analysis from available data. Written informed consent for participation was not obtained from the owners because the production and medical data used in this study are from farm sites that have a management contract with Pipestone. This contract gives Pipestone permission to use and access the information. No site is identifiable from the data and analysis in this work.

## Author contributions

KH, GS, and JN conceptualized the study. KH performed the analysis. LB and RC compiled the data used and gave guidance on its interpretation. SD and JN provided guidance on Porcine reproductive and respiratory syndrome virus (PRRSV) sequencing classifications. KH and SD developed the manuscript. SD developed and tested the biosecurity protocols described in the manuscript. All authors contributed to the manuscript revision and read and approved the submitted version.
